# Point-of-care bone cement based on natural peptide comonomer protects orthopaedic implants from bacterial challenge

**DOI:** 10.1016/j.mtbio.2025.102388

**Published:** 2025-10-07

**Authors:** Yang Xu, Hao Lin, Yin-Yu Qi, Chen Wang, Long-Xu Han, Fang He, Hong-Xun Sang, Jian-Jun Chu

**Affiliations:** a School of Food and Biological Engineering, Hefei University of Technology, Hefei, China; b The Second People's Hospital of Hefei, Anhui Medical University, Hefei, China; c Hefei BOE Hospital, Shanghai University Medical College, Hefei, China; d Shenzhen Hospital, Southern Medical University, Shenzhen, China

**Keywords:** Polymethylmethacrylate (PMMA) bone cement, Periprosthetic joint infection (PJI), Nisin, Point-of-care (POC), Natural peptide comonomer

## Abstract

Antibacterial polymethylmethacrylate (PMMA) bone cement plays an important role in the prevention of periprosthetic joint infection (PJI). PMMA cement based on the immobilization of antibacterial comonomers has expanded the horizon of the PJI prophylaxis, but creating clinically-convenient and accessible cement remains challenging. Here, we have developed a nisin based point-of-care (POC) technology. This technology relies on the synergistic engineering of the natural peptide nisin and can be applied in the operating room. Nisin can act as a comonomer and be immobilized via naturally occurring dehydroalanine (Dha), thereby endowing the cement with antibacterial activity (up to 98 %), mechanical strength (compliant with ASTM F451 standard, minimum 70 MPa), as well as biocompatibility both *in vitro* and *in vivo*. The utility is further exemplified by a periprosthetic infection model in rats, with compatibility studies confirming its seamless integration with commercially available cements. All the raw materials are commercially available and easily accessible, and the usage process is consistent with that of clinical cement, showing excellent operability, thus expanding the toolbox for PJI prophylaxis.

## Graphical abstract

A point-of-care technology based on the natural peptide comonomer Nisin has been reported. This technology can combine with existing PMMA bone cement, enabling surgeons to conveniently prepare antibacterial bone cement–which features no burst release, excellent mechanical properties, and good biocompatibility–without specialized training.

**Figure:**
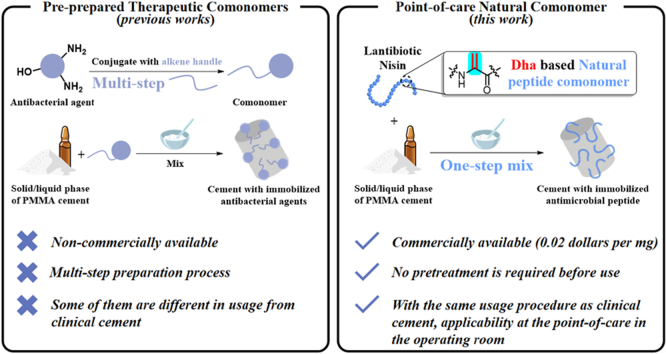

## 1 Introduction

Driven by factors such as global population aging and warfare trauma, millions of total joint arthroplasty (TJA) procedures are conducted annually [[1], [2], [3]]. Periprosthetic joint infection (PJI) represents a severe complication of TJA and ranks among the most prevalent causes for revision surgeries [4,5]. The 5-year mortality rate subsequent to PJI ranges approximately from 20 % to 30 %, surpassing that of numerous cancers, thereby imposing a substantial medical burden [3]. To mitigate the risk of PJI, antibiotic-loaded PMMA bone cement (ALBC) is extensively employed for prophylactic measures [[6], [7], [8], [9], [10], [11]]. Prophylactic ALBC is engineered to uphold the long-term integrity of implants over their entire service life. After decades of research and application, ALBC has been found to exhibit several drawbacks, including high polymerization temperature, shrinkage after polymerization, suboptimal drug release behavior, etc. Among these characteristics, the antibiotic release behavior of ALBC represents a particularly noteworthy limitation: antibiotics typically undergo a rapid burst release within the initial few hours post-implantation. High-dose antibiotics not only exhibit cytotoxicity and impede osteogenic activity but, more critically, the burst release restricts the effective antibiotic release to merely one week. Subsequently, the residual antibiotics prove inadequate to suppress or eradicate bacteria, which readily promotes the development of bacterial resistance and fails to offer optimal protection against infections [[12], [13], [14], [15], [16], [17], [18]]. Furthermore, after the antibiotic clusters are eluted from ALBC, numerous voids form, causing the compressive strength to drop below the minimum threshold of 70 MPa specified by ASTM standards and thus necessitating a second surgery [19,20]. These limitations undermine the efficacy of ALBC in preventing infections [11,13,20].

**Fig. 1:**
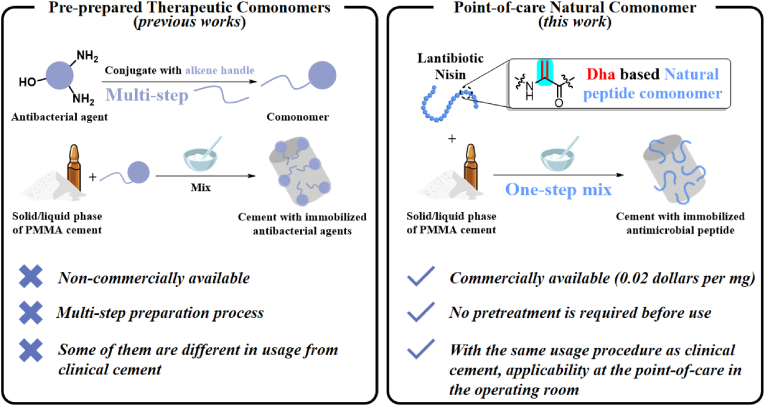
**Approaches for fabricating antibacterial PMMA bone cement using comonomers.** Generic Diagramming Platform (https://biogdp.com/) was used to draw schematic diagrams.

Recently, reported bone cements with immobilized antibacterial agents avoid burst release and exhibit good mechanical strength. A typical one uses a therapeutic methacrylic (TMA) comonomer [20] (Fig. 1), and antibiotics like gentamicin and isoniazid can be chemically modified into comonomers [[20], [21], [22], [23]]. Also, many alkene-functionalized antibacterial motifs are also comonomers that can fabricate such bone cements [[24], [25], [26], [27], [28]]. However, despite their contributions, the operational procedures are complex. Comonomer preparation typically involves multi-step synthesis and purification. These procedures have raised barriers for clinical researchers to explore and adopt this technology. To address this challenge, we endeavored to design a bone cement preparation technology that can be applied at the point-of-care (POC) in the operating room and does not require alteration of the cement manufacturing process.

Lantibiotics, natural antimicrobial peptides (AMPs) also called bacteriocins, feature diverse structures, low cost, and easy availability, with wide use in pharmaceuticals and food industries [[29], [30], [31], [32]]. For example, they act as lactobacillin tablets for gastrointestinal infections and the preservative nisin. Lantibiotics form cyclic structures via thioether bonds between dehydroalanine (Dha)/dehydrobutenine and cysteine, ensuring stability [[33], [34], [35]]. A careful examination of lantibiotic's structures has surprisingly revealed that many lantibiotics, such as nisin, contain Dha in unbonded forms-a versatile non-canonical amino acid that offers their potential as natural peptide comonomers. Since high temperatures are generated during the preparation of PMMA cement, the stability of lantibiotics further enhances the promise of their success as comonomers.

In this study, we used commercially available, inexpensive, and safe lantibiotic nisin to investigate novel antibacterial bone cement (Fig. 1). We aim to assess the therapeutic efficacy, functionality, safety, and operability of nisin based cement. We demonstrate that nisin can be used to prepare high-performance antibacterial bone cement in an extremely simple and effective manner. This cement exhibits nearly 99 % antibacterial activity, excellent mechanical strength, high biocompatibility, and applicability at the point-of-care in the operating room.

We believe that this breakthrough discovery, although the published method is still in the developmental stage (largely because numerous critical factors, including the mixing process, the presence of naturally occurring contaminants in the surgical field (e.g., blood, bone fragments, saline), and minor deviations in the proportions of the binary components, have not yet been considered, and all these factors exert a key influence on the final performance of bone cement) and requires optimization—holds great significance for preventing infection [[36], [37], [38], [39], [40], [41]]. The natural peptide conomoner-based technology, from a translational perspective, is compatible with the solidification of bone cement and dental resin, and thus may be applied as a prophylactic standard care to prevent implant-associated osteomyelitis. Although the manufacturing cost of this POC technology is low, since it is used to produce medical devices—products that involve complex certification procedures and strict regulatory requirements—further in-depth research is still needed for its practical translation into clinical applications. We sincerely hope that this discovery will inspire further exploration and warmly welcome all interested researchers from academic and medical communities to contact and collaborate with us, so as to jointly advance this promising technology.

## 2 Materials and methods

### 2.1 Materials

Polymethyl methacrylate (PMMA), methacrylic acid (MMA), 2 %ARS working solution, polyvinylidene fluoride (PVDF) membrane and chemiluminescence kit were purchased from Sigma Aldrich, St. Louis, USA. Benzoyl peroxide (BPO), N, N-dimethyl-p-toluidine (DMPT) were purchased from J&K Scientific Ltd, China. BaSO_4_ and Cetylpyridine chloride solution were purchased from Shanghai Aladdin Bio-Chem Technology Co., Ltd. Heraeus bone cement (OSTEOPAL®V) was purchased from Heraeus Medical GmbH. Eurofix bone cement was purchased from Shanghai Lenovo Medical Co., Ltd. Nisin was purchased from Guangzhou Muran Biochemical Technology Co., Ltd. Gentamicin sulfate (GS) was purchased from Macklin Biochemical Technology Co., Ltd, China. 2.5 % glutaraldehyde (for electron microscopy) was purchased from Shanghai Acmec Biochemical Technology Co., Ltd. Zoletil®50 was purchased from Guangzhou Furatge Biotechnology Co., Ltd. *Staphylococcus aureus* (*S. aureus,* ATCC 25923) and clinical isolated multidrug-resistant *S. aureus* (hereinafter referred to as clinical MRSA, this strain is resistant to a variety of carbapenem antibiotics and resistant to gentamicin, see Supplementary Information Table S1 for details) were obtained from the Department of Clinical Laboratory, Hefei Second People's Hospital. MC3T3-E1 cells and their specialized cell culture medium were purchased from Wuhan Servicebio Biotechnology Co., Ltd. Human bone marrow mesenchymal stem cells (hBMSCs) and hBMSC-specific medium were purchased from iCell Bioscience Inc, Shanghai. MTT reagent was purchased from Solarbio Scinence & Technology Co., Ltd, Beijing, China. ALP assay kit, Trizol reagent, PMSF and BCA protein quantification kit were purchased from Beyotime Biotechnology Co., Ltd, Shanghai, China. dNTP reagent and cDNA synthesis kit were purchased from Takara, Japan. RNase-free H2O was purchased from Coolaber, China. SDS-PAGE gel kit was purchased from EpiZyme Biotechnology Co., Ltd, China. Anti-ALP (1:1000) was purchased from Affinity, T0023, USA. Anti-RUNX2 (1:1000) was purchased from Cell Signaling Technology, #12556, USA. SPF grade C57 mice, SD rats and rabbit erythrocytes were provided by the Animal Experiment Center of Anhui Medical University. BPO is vacuum-dried at room temperature for 24 h before use. PMMA is ground to a particle size of 80–100 mesh and used. Other chemicals do not require further purification and can be used directly.

### 2.2 Preparation of bone cement

All self-made bone cements with different formulations used in this study were prepared according to the formulas listed in Table 1, and their formulation design was close to that of commercially available bone cements. For the preparation of commercially available bone cements, the amount of drug added is the corresponding percentage relative to the mass of the solid phase. Then the solid and liquid phases of the bone cements were uniformly mixed at room temperature, they were filled into PTFE molds of different specifications for molding. After curing and demolding, they were used for various experimental studies.

**Table 1:** Bone cement formulations. (The total amount of bone cement is approximately 1 g).

Formulation	Powder (mg)				Liquid (μL)	
	PMMA	BPO	BaSO_4_	Drug	MMA	DMPT
PMMA cement	525	13	100	0	379	7.5
2.5 % Nisin cement	509.05	13	100	15.95	379	7.5
5 % Nisin cement	493.1	13	100	31.9	379	7.5
10 % Nisin cement	461.2	13	100	63.8	379	7.5
5 % GS cement	493.1	13	100	31.9	379	7.5

### 2.3 Mechanical strength

Cylindrical (Ø6 × 12 mm) bone cements, including PMMA, 2.5 % Nisin, 5 % Nisin, and 10 % Nisin cements, were prepared. For each type, five samples were soaked in normal saline at room temperature for 7 days, while the others were not soaked. The bone cements were polished with 1000-mesh sandpaper to ensure their upper and lower surfaces were parallel, and their weight was controlled at 0.4 ± 0.01 g. Compression tests were conducted using a computer-controlled material testing machine (BOSE Corporation, USA) at 23 °C with a loading rate of 20 mm/min until the samples fractured. The compressive strength was determined as the maximum value on the stress-strain curve, and the elastic modulus was calculated from the linear slope of the stress-strain curve. The mechanical strength of each group of bone cement was evaluated and analyzed in accordance with ASTM standards. Five samples (n = 5) were tested for each group of bone cement.

### 2.4 Fourier transform infrared spectroscopy (FT-IR)

A small amount of bone cement powder was mixed with potassium bromide at a ratio of approximately 1:50, then ground and pressed into sheets. These mixtures were analyzed via FT-IR spectroscopy using a BRUKER VECTOR-22 spectrometer, over the frequency range of 4000-400 cm^−1^.

### 2.5 Scanning electron microscopy (SEM)

Each group included two square flake samples (20 mm × 20 mm × 2 mm) of PMMA, 2.5 % Nisin, 5 % Nisin, 10 % Nisin, and 5 % GS bone cements. For each type, one sample was first subjected to liquid nitrogen brittle fracture and then soaked in normal saline at room temperature for 7 days, while the other was not soaked. The treated bone cements were coated with gold particles using a sputter coater, and the cross-sectional morphology of the bone cements before and after the 7-day soaking was characterized using SEM (ZEISS Sigma 360, Germany).

Square flake samples (5 mm × 5 mm × 2 mm) of PMMA cement, 2.5 %, 5 %, 10 % Nisin cements were prepared. After soaking in normal saline for 1 day, each group of bone cements (sterilized by UV irradiation for 30 min) was placed in a sterile glass tube, and 1 mL of the aforementioned *S. aureus* bacterial suspension was added to each tube. The glass tubes were then incubated in a 37 °C, 5 % CO_2_ incubator for a total of 6 h. Then the cements were removed and transferred to sterile centrifuge tubes, followed by gentle rinsing three times with 100 mL of double-distilled water to remove planktonic bacteria. Subsequently, the cements were transferred to 2 mL centrifuge tubes, and 1.5 mL of 2.5 % glutaraldehyde solution was added; they were then incubated overnight in a 4 °C refrigerator. The treated bone cements were coated with gold particles using a sputter coater, and the bacteria adhering to their surfaces were characterized using a SEM (ZEISS Sigma 360, Germany). The morphology and distribution of the bacteria were observed.

### 2.6 Water contact angle

In order to evaluate the hydrophilic properties of the bone cements, square flake samples (20 mm × 20 mm × 2 mm) of PMMA, 2.5 % Nisin, 5 % Nisin, and 10 % Nisin bone cements were prepared. The samples were placed on the test platform of a JY-82C video contact angle meter. Water droplets were dispensed via the instrument's automatic titration system, test images were captured, and the water contact angle was measured using the high-volume method (specifically, lines are drawn at the intersection of the sample surface and the droplet in the image, and the resulting angle is the contact angle). Three samples were tested for each group of bone cements.

### 2.7 Surface roughness

A Bruker Dimension ICON atomic force microscope (AFM) was used to measure the nanoscale surface roughness of the bone cement samples. Three scanning areas of 5 mm × 5 mm were selected on each sample, and NanoScope analysis software was employed to perform three-dimensional parameter analysis of the AFM images, in order to evaluate surface roughness variations among different samples. For each group of samples, three measurement areas were analyzed, and the average value was taken as the final result.

### 2.8 *In vitro* mineralization

The simulated body fluid (SBF) immersion method was employed to evaluate hydroxyapatite formation and verify the *in vitro* biomineralization activity of the samples. Square flake samples (20 mm × 20 mm × 2 mm) of PMMA, 2.5 % Nisin, 5 % Nisin, and 10 % Nisin bone cements were prepared. Bone cements from different groups were soaked in SBF at a ratio of 0.1 cm^2^ (sample surface area) per cm^3^ (SBF volume) and then incubated at a constant temperature of 37 °C for 7 days. After incubation, the samples were washed and dried at 25 °C. Subsequently, scanning electron microscopy (SEM) and energy dispersive spectroscopy (EDS) point analysis were used to observe the surface topography of the materials and the formation of hydroxyapatite.

### 2.9 High-performance liquid chromatography (HPLC)

The *in vitro* release characteristics of Nisin cements were investigated using high-performance liquid chromatography (HPLC). Each bone cement was prepared into cylindrical specimens (Ø6 × 12 mm), which were subsequently immersed in 3 mL of deionized water for 24 h. A 200 μL aliquot of the extract was analyzed by HPLC, and the components were identified using electrospray ionization mass spectrometryry (ESI-MS). HPLC conditions: column: C18 (4.6 × 250 mm); solution A was 0.1 % trifluoroacetic acid (TFA) in water, and solution B was 0.8 % TFA in MeCN; gradient: a linear gradient of 1 % to 90 % B over 30 min; flow rate 1.0 mL min^−1^; UV–Vis detector: 214 nm.

### 2.10 Antibacterial activity

*S. aureus* and clinical MRSA were resuscitated and subcultured. Bacteria that had undergone three subcultures were used to prepare bacterial suspensions with a concentration of 0.5 × 10^8^ CFU/mL, which were set aside. Disc-shaped bone cements (Ø6 × 3 mm) were prepared, including PMMA cement, 2.5 %, 5 %, 10 % Nisin cement. A 100 μL aliquot of the *S. aureus* suspension was pipetted and evenly spread over nutrient agar plates. The bone cements (sterilized by UV irradiation for 30 min) were then placed on the plates and labeled. The plates were incubated in a 37 °C, 5 % CO_2_ constant-temperature incubator for 24 h, and the size of the inhibition zones was observed to evaluate the antibacterial activity of the bone cements via released Nisin against *S. aureus*. Additionally, 2.5 %, 5 %, and 10 % GS bone cement was tested for their inhibition zones against clinical MRSA to assess their antibacterial activities. For each group, three cylindrical bone cements (Ø6 × 12 mm) were prepared, including PMMA cement, 2.5 %, 5 %, 10 % Nisin cement. These cements were soaked in normal saline for 1, 7, and 14 days, respectively. After soaking, the samples were sterilized by UV irradiation for 30 min, then each cylindrical bone cement was placed in a sterile glass tube, and 1 mL of the aforementioned *S. aureus* suspension was added to each tube. The glass tubes were incubated in a 37 °C, 5 % CO_2_ incubator for a total of 6 h. After 6 h, the cements were removed and transferred to sterile centrifuge tubes, then gently rinsed three times with 100 mL of double-distilled water to remove planktonic bacteria. Subsequently, the bone cements were transferred to centrifuge tubes containing 5 mL of normal saline and sonicated for 3 min using an ultrasonic cleaner. After sonication, 40 μL of the sonicated solution was diluted in 4 mL of normal saline. Following thorough mixing, 40 μL of the diluted solution was pipetted for plate coating. The coated nutrient agar plates were incubated in a 37 °C, 5 % CO_2_ incubator for 24 h. After 24 h, colony growth was observed and counted, and the inhibition rate was calculated. Three samples (n = 3) were tested for each group of bone cements.

### 2.11 Hemolysis assay

Cylindrical (Ø6 × 12 mm) PMMA, 2.5 %, 5 %, and 10 %Nisin cements were prepared. The bone cement was soaked in normal saline for 24 h. The soaked bone cement (sterilized by UV irradiation for 30 min) was put into a sterile centrifuge tube, and normal saline was added at a sample and liquid ratio of 0.2 g/mL according to the GB/T 16886 standard, and incubated in an incubator at 37 °C and 5 % CO_2_ for 30 min 1.8 mL of the extract of each group was placed in a sterile centrifuge tube, 1.8 mL of purified water was taken as the positive control, and 1.8 mL of normal saline was used as the negative control. Add 200 μL of 2 % rabbit erythrocyte suspension to each tube. The tubes were then incubated at 37 °C with 5 % CO_2_ for 1 h, followed by centrifugation of the solutions at 3,000 rpm for 5 min. Visually inspect the transparency of the supernatant to assess for hemolysis. Measure the optical density (OD) of the supernatant at 545 nm using a microplate reader. Three samples (n = 3) were measured in each group of bone cement. The hemolysis rate (HR) is calculated according to the following formula:

$$HR=\frac{{OD}_{T1}-{OD}_{N1}}{{OD}_{P}-{OD}_{N1}}\times 100\%$$

OD_T1_ is the optical density of the experimental solution, OD_N1_ is the optical density of the negative control, and OD_P_ is the optical density of the positive control. A hemolysis rate of less than 5 % is considered to be hemolysis-free.

### 2.12 CCK-8

Cylindrical (Ø6 × 12 mm) PMMA, 2.5 % Nisin, 5 % Nisin, 10 % Nisin bone cements were prepared. The bone cement was soaked in normal saline for 24 h, and then the soaked bone cement (sterilized by UV irradiation for 30 min) was put into a sterile centrifuge tube, and the special medium for mouse embryonic osteoblast precursor cells (MC3T3-E1) was added at a sample to liquid ratio of 0.2 g/mL according to GB/T 16886 standard, and placed in an incubator at 37 °C and 5 % CO_2_ for 24 h to prepare bone cement cell culture medium extract.

Quickly thaw the vial containing mouse embryonic osteoblast precursor cells (MC3T3-E1) in a water bath at 37 °C until the solution in the tube is completely thawed. Thawed cells were centrifuged, resuspended, and cultured in a sterile environment. Cells were cultured at 37 °C and 5 % CO_2_ using MC3T3-E1 cell-specific medium, and cells that underwent 3 subcultures were selected for experimentation. Cells in the logarithmic growth phase were trypsinized, centrifuged and counted, and then cells were seeded into 96-well plates with approximately 5000 cells per well and continued in culture medium. After the cells were attached, the original medium was discarded and the extract of bone cement medium prepared above was added, 100 μL per well, and co-cultured with MC3T3-E1 cells.

Mix CCK-8 reagent with culture medium at a volume ratio of 1:9 to prepare a mixed solution. After 1day, 3 days, and 5 days of cell co-culture, the medium in the wells was discarded, 100 μL of the above mixed solution was added, and the incubation was continued for 2 h, and the morphological changes of the cells were observed using an inverted fluorescence microscope (Nikon DS-Fi3). Measure the optical density (OD) of each well using a microplate reader at a wavelength of 450 nm, and calculate the relative growth rate (RGR) of cells in each group using the formula below. The morphological changes and cytotoxicity of cells were evaluated according to GB/T16886 standard. Three samples (n = 3) were measured in each group of bone cement.

$$RGR=\frac{{OD}_{T2}-{OD}_{R}}{{OD}_{N2}-{OD}_{R}}\times 100\%$$

OD_T2_ is the optical density of the experimental group; OD_N2_ was the optical density of the blank control group. OD_R_ is the optical density of cell-free media.

### 2.13 Cell culture

Human bone marrow mesenchymal stem cells (hBMSCs) were cultured in non-serum culture system of primary mesenchymal stem cells. The cells were maintained at 37 °C in a 5 % CO_2_ incubator. Prior to the start of the experiment, all bone cement samples were subjected to high-temperature and high-pressure steam sterilization (121 °C, 20 min).

### 2.14 Cellular compatibility and proliferation

Cylindrical (Ø10 × 3 mm) PMMA, 2.5 %, 5 %, and 10 %Nisin bone cement were prepared. hBMSCs (6 × 104 cells/well) were added onto bone cement samples in 12-well plates and incubated at 37°Cin a 5 % CO_2_ environment. The 1000 μl of MTT reagent (0.5 mg/ml) to each well was added at 1 day, 3 days, and 5 days, respectively, and the plates were further incubated at 37 °C in a 5 % CO_2_ environment for 4 h. Aspirate the supernatant, add 1000 μl DMSO to each well, shake gently for 10 min, then transfer 100 μl from each well to a 96-well plate. The absorbance of the reaction solution was measured at 570 nm using a microplate reader.

### 2.15 Osteogenic differentiation

Cylindrical (Ø15 × 3 mm) PMMA, 2.5 %, 5 %, and 10 %Nisin bone cement were prepared. The osteoblastic differentiation of hBMSCs was evaluated by alkaline phosphatase (ALP) staining and Alizarin Red S (ARS) staining. hBMSCs (2 × 10^5^ cells/well) were added to bone cement samples in 6-well plates and incubated at 37 °C in a 5 % CO_2_ environment. When the confluence of hBMSCs reached 80 % or more, the bone cement samples were transferred to the upper chamber and cultured with 2 mL of osteogenesis-inducing medium containing β-glycerol phosphate (10 mM), L-ascorbic acid (50 μM), and dexamethasone (0.1 μM). On the 7th day, 500 μL of 4 % para formaldehyde was added to fix the cells for 20 min. According to the ALP assay kit instructions, 300 μL of the mixture was added, and the samples were incubated at room temperature for 12 h. On the 14th day, after fixing the cells, 300 μL of 2 % ARS working solution was added for 15 min. All samples were observed and photographed under an inverted microscope. Subsequently, cetylpyridine chloride solution was added, and the samples were incubated at room temperature in the dark for 30 min. Finally, the absorbance of the mixture was measured at 562 nm using a microplate reader.

### 2.16 qRT-PCR

Cylindrical (Ø15 × 3 mm) PMMA, 2.5 %, 5 %, and 10 %Nisin bone cement were prepared. After 14 days of osteogenic culture, the total RNA was extracted from hBMSCs in different groups by adding Trizol reagent, and reversely transcribed into cDNA using a synthesis kit. The gene levels of ALP, Runx2, were analyzed by qRT-PCR using a mixture of dNTP reagent and RNase-free H2O, as well as the forward and reverse primers listed below. The 2−ΔΔCT method was used to analyze gene expression data and (β-actin) gene was employed as the housekeeping gene for normalization. The primer sequences used for the genes are presented in Table S2.

### 2.17 Western blot

Cylindrical (Ø15 × 3 mm) PMMA, 2.5 %, 5 %, and 10 %Nisin bone cement were prepared. The cells were lysed using RIPA lysis solution containing the protease inhibitor PMSF. The BCA protein quantification kit was used to determine the concentration of total protein. According to the SDS-PAGE gel kit instructions, the proteins were separated using a voltage of 110 V. Then, the protein was transferred to a polyvinylidene fluoride (PVDF) membrane using a constant current of 300 mA. The PVDF membrane was blocked with 5 % milk at room temperature for 1.5 h. The membrane was then incubated with anti-ALP (1:1000) and anti-RUNX2 (1:1000) antibodies at 4 °C over-night. The next day, the membrane was washed with TBST and incubated with the secondary antibody at room temperature for 1 h. The blots were detected using a chemiluminescence kit Semi-quantitative analysis was performed using Image J software (National Institutes of Health, USA).

### 2.18 *In vivo* evaluation of bone cements

#### 2.18.1 Acute toxicity test in mice

Twenty-five C57 mice weighing in the range of 17–21 g were selected and divided into 5 groups of 5 in each group according to a completely random grouping method. At a distance of 5 mm from the white line of the lower abdomen of mice, the normal saline extract of each group of bone cement prepared in the above way was injected into the abdominal cavity of mice through the intraperitoneal cavity at a ratio of 50 mL/kg, and raised in a ventilated and dry environment at 18–22 °C, and sufficient food and water were given. At 24 h, 48 h and 72 h after injection, the general condition and toxicity of the mice were observed, and the body weight changes of the mice were recorded. At 72 h, the mice were euthanized with cervical dislocation, and the liver and kidney were stained with hematoxylin-eosin and observed under a microscope.

#### 2.18.2 Rat back muscle implantation

Six SD rats weighing in the range of 290–310 g were selected and skinned 1 day before operation. The anesthetic drug (Zoletil®50) was injected into the rat leg muscle at a dose of 50∼75 mg/kg, and disinfect and draping in strict accordance with the surgical requirements before surgery. Select two implant sites approximately 1 cm from the spine on both side of the rat's back, with a distance of approximately 2.5 cm between the upper and lower sides of each side. The skin fascia was incised, the muscle tissue was bluntly separated along the long axis to form a muscle cavity, and PMMA and 5 % Nisin bone cement (disc (Ø6 × 3 mm) bone cement sample) were implanted, two on each side, and then the incisions were sutured in layers. Two rats were sacrificed at 2, 4 and 8 weeks after surgery, and cut the muscle tissue around the bone cement was about 0.5 cm. Observe the local infection (hematoma, suppuration, etc.) and material displacement with the naked eye. Muscle tissue was sectioned, stained with hematoxylin-eosin and observed under a light microscope.

#### 2.18.3 Rat femur implant infection

Cylindrical (Ø1 × 6 mm) PMMA and 5 %Nisin bone cement were prepared, and after sterilization by UV irradiation, they were cultured in the above-mentioned *S*. *aureus* bacterial solution for 6 h. Nine SD rats weighing in the range of 290–310 g were selected and completely randomly divided into 3 groups of 3 rats in each group. Prepare the skin 1 day before surgery. The anesthetic drug (Zoletil®50) was injected into the muscle of the right leg of the rat with an injection volume of 50∼75 mg/kg, and disinfect and draping in strict accordance with the surgical requirements before surgery. The skin of the rat's knee joint was cut along the long axis, and the patella, muscle, and ligament were bluntly separated, exposing the articular surface of the distal femur. Use a suitable-sized K-wire to drill a suitably sized cavity along the long axis of the femur, and fill the hole with bone cement attached to the bacterial fluid, and the control group only drills the hole. The implant hole was sealed with bone wax, and replaced the muscular ligaments and patella, cleaned the wound, sutured layer by layer, and bandaged. Starting on the 3rd postoperative day, an appropriate amount of jugular venous blood was drawn every 3 days for white blood cell count. After 2 weeks, all rats were sacrificed, the healing of the surgical wound and the infection were observed, and X-rays were taken to observe the implantation of bone cement. The implanted bone cement was removed and an appropriate amount of tissue around the wound was incised for bacterial quantification, the liver and kidney of the rat were removed, and the liver and kidney were stained with HE after fixation, and sections were made to observe the toxicity of the liver and kidney.

### 2.19 Nisin-based POC technology in clinical cement

In order to further explore the clinical operation performance of Nisin bone cement and the performance of Nisin in commercially available bone cement, operability experiments were performed on knee prostheses using Heraeus cement. Heraeus and Eurofix bone cement were also used to prepare PMMA and 5 % Nisin bone cement for surface antibacterial experiments (same as above), determination of setting time and maximum temperature, and determination of mechanical properties (same as above).

The setting time and maximum temperature determination method are as follows: under the condition of 24 ± 1 °C, 1 g of bone cement is filled into the mold, and the temperature change with time in the polymerization process is recorded by infrared thermal imager. The real-time temperature is recorded every 15 s to calculate the operating temperature to determine the setting time. The solidification time is calculated using the formula T_set_ = (T_max_ + T_amb_)/2, where T_max_ is the maximum polymerization temperature, T_amb_ is the ambient temperature, and t_set_ is the solidification time corresponding to T _set_. Prepare 3 identical bone cement samples for each group, and replicate the entire experiment 3 times (n = 3).

### 2.20 Statistical analysis

The data in this paper were analyzed using SPSS27.0 software, the measured values were mean ± standard deviation, and the antibacterial performance, compressive strength, elastic modulus, hemolysis rate, coagulation time, and polymerization temperature were statistically analyzed using univariate analysis (ANOVA). Relative growth rate (RGR) was assessed using repeated measures ANOVA. Statistically significant was defined as a *p*-value of less than 0.05 (*p* < 0.05).

## 3 Results and discussion

### 3.1 Preparation and characterization of Nisin based bone cement

Nisin is a natural antibiotic containing two unbonded Dha residues (Fig. S1A). Commercially available nisin contains 2.5 % peptide and mainly exists in two types, A and Z, which differ by only one amino acid residue and have similar activities. In this study, we used nisin Z, and we use "Nisin" to refer to commercially available nisin. Given that PMMA polymerization is exothermic, we heated nisin under simulated exothermic conditions and evaluated its structural stability during polymerization by comparing the retention time and peak shape in its chromatograms before and after heating. (Fig. S1B). After confirming that the heat released during PMMA polymerization does not affect the structure of Nisin, we prepared four types of bone cements: bare PMMA cement without any drug addition, and three bone cements containing different Nisin contents, namely 2.5 % Nisin cement, 5 % Nisin cement, and 10 % Nisin cement (Table 1). Similar to the preparation of ALBC, we first mixed Nisin uniformly with the solid phase of the bone cement, then added the liquid phase, stirred for a period, and used molds to form specimens of different shapes.

**Fig. 2:**
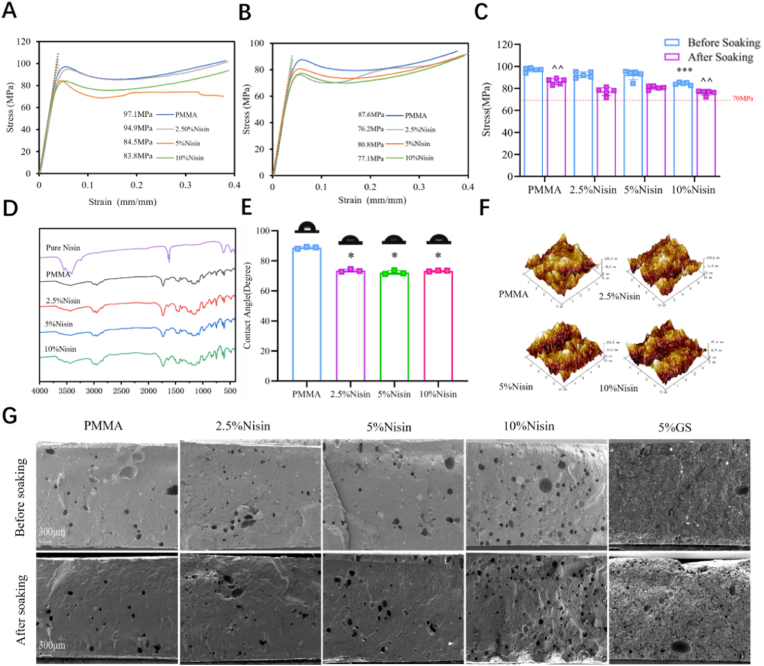
(A, B) The stress-strain curves of PMMA, 2.5 %, 5 % and 10 % Nisin cement before and after soaking for 7 days, and the data below the curve are compressive strength. (C) The compressive strength before and after soaking for 7 days (∗∗∗: p < 0.01, compared to the other three groups; ^^: p < 0.01, comparison between the two groups. (D) FT-IR of Nisin, PMMA cement, 2.5 %, 5 % and 10 % Nisin cement. (E) Water contact angle of PMMA cement, 2.5 %, 5 % and 10 % Nisin cement (∗: p < 0.01, compared to PMMA). (F) Surface roughness of PMMA cement, 2.5 %, 5 % and 10 % Nisin cement. (G) SEM of PMMA cement, 2.5 %, 5 %, 10 % Nisin cement and 5 % GS cement before and after soaking for 7 days.

Bone cement serves as an adhesive for fixing joint prostheses, and the ASTM standard requires its compressive strength to exceed 70 MPa. As shown in Fig. 2A, we found that the compressive strength of both PMMA cement and Nisin cements exceeded 70 MPa. Since the mechanical properties of bone cement may decline after implantation, we investigated the compressive strength of aged bone cements (Fig. 2B). The results showed that, similar to PMMA cement, the compressive strength of Nisin cements decreased slightly after aging but remained above 70 MPa (Fig. 2C). Fig. S2 shows the changes in elastic modulus before and after aging. Fig. 2D presents Fourier transform infrared (FT-IR) spectra of PMMA cement and Nisin cements, which had the same signal peaks at 2950, 1730, 1244, and 1150 cm^−1^ that represented the characteristic peaks of the acrylic bone cement. FT-IR analysis revealed a broad amide I band at 3400 cm^−1^ (attributed to *ν* N-H stretching vibration), along with amide II (*ν* C=O stretching vibration) and amide III (*ν* N-H stretching vibration and *δ* N-H bending vibration) bands at 1650 cm^−1^ and 1550 cm^−1^ respectively; however, the peptide content in commercial Nisin is only 2.5 %, resulting in indistinct peaks in the FT-IR spectra of both Nisin and Nisin cements. The wettability and surface roughness of cements were further characterized by water contact angle measurement and atomic force microscopy (AFM), respectively. As shown in Fig. 2E, the water contact angle analysis on PMMA cement and Nisin cements revealed significant contact angle changes. The contact angle reduced from 89° to 73° after the hydrophilic peptide comonomer Nisin incorporation. The surface roughness of the cement slightly decreased after the addition of Nisin. The bare PMMA cement exhibited a relatively smooth surface morphology with a roughness (Ra) of 32.7 ± 11.3 nm. After Nisin modification, the roughness showed a trend of slight decrease followed by an increase, but the overall change was minimal.

We also characterized the fracture surfaces of PMMA cement, Nisin cements and GS cement (traditional gentamicin-loaded bone cement) using scanning electron microscopy (SEM). The fracture surfaces of both PMMA cement and Nisin cements were smooth, with some circular pores distributed across them (Fig. 2G). These pores are air bubbles entrained during bone cement preparation, which can be observed after the bone cements are brittle-fractured in liquid nitrogen. As the Nisin addition amount increased, the number of bubbles increased, primarily due to the need for longer stirring times to promote solidification as the additive amount increased, thereby entraining more air bubbles. This contributed to the lower mechanical strength of 10 % Nisin cement, although it still remained higher than 70 MPa. Additionally, we used SEM to examine Nisin cements fracture surfaces after soaking in water for 7 days, and compared to the unsoaked cements, almost no changes were visible. This is consistent with previously reported characteristics of bone cements with antibacterial comonomers before and after soaking [20,23]. With the increase in Nisin content, the number of bubbles in the cement increases. This change in microstructure also matches the decrease in mechanical properties caused by the increase in Nisin content. For traditional ALBC, such as the gentamicin formulation, the fracture surface typically produces many voids formed by the elution of drug clusters after soaking (Fig. 2G). It is more obvious that some voids are formed in the fracture surface of the bubbles after soaking. These are formed after the drug clusters are eluted. In Nisin cements, the voids formed by the elution of drug clusters were not found after soaking (Figs. S3–S7). All these indicated the successful incorporation of the Nisin commoner into cement.

### 3.2 Antibacterial activity *in vitro*

**Fig. 3:**
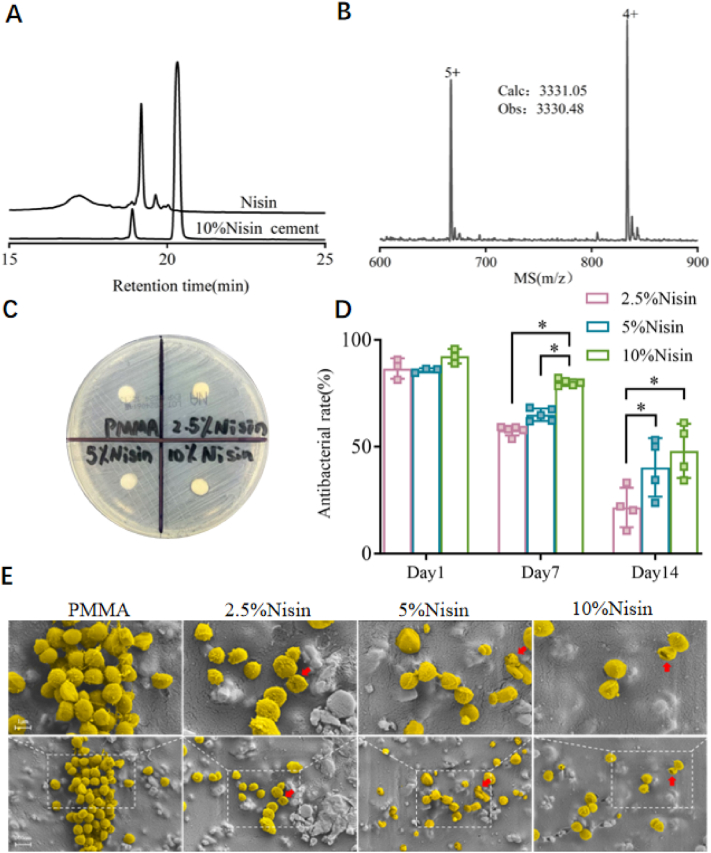
(A) HPLC of Nisin and 10 % Nisin cement extract. (B) ESI-MS of Nisin. (C) Inhibition zone of bone cements against *S. aureus*. (D) Antibacterial rate of Nisin cements against *S. aureus* within 14 days (∗: p < 0.01). (E) SEM images of *S. aureus* on different cement surfaces. The *S. aureus'* in the figure are labeled in golden yellow, and the cytomembrane damage sites are indicated by red arrows.

The leachate of bone cement was detected by HPLC to confirm whether the comonomer in Nisin cements was leached out. As shown in Fig. 3A, no Nisin was leached from the cement. Fig. 3B presents the mass spectrum of commercially available Nisin. The cement itself exhibited several peaks in the HPLC, which were confirmed by mass spectrometry not to be Nisin (Fig. S8). The bacteriostatic ring test of the cements showed that Nisin did not diffuse antibacterial substances (Fig. 3C). These results indicate that Nisin cement does not release Nisin comonomer, and this conclusion consistent with the unchanged microstructure observed by SEM before and after soaking. This characteristic differs from traditional ALBC, whose antibacterial activity relies on the dissolution and subsequent leaching of drug clusters in the cement by water. We investigated the antibacterial activity of the bone cements, and the bacteria were cultured on different cement samples for 6 h, then harvested and plated on agar plates. Compared with bare PMMA cement, the antibacterial activity of Nisin cements was significantly enhanced (Fig. 3D). The antibacterial activity of Nisin cements was proportional to the amount of comonomer. The antibacterial rate of 2.5 % and 5 % Nisin cements both exceeded 90 %, and the antibacterial rate of 10 % Nisin cement almost reached 100 %. Due to the burst release, ALBC cannot release antibiotics above the MIC value one week after implantation, thus losing its antibacterial activity [12,13,42,43]. To study the change in antibacterial activity of Nisin cements after implantation, we conducted 7-day and 14-day aging tests. The antibacterial activity of Nisin cements decreased with the prolongation of aging time. It is exciting that the antibacterial activity of the cements was well maintained, especially for 5 % and 10 % Nisin cements. We were surprised to find that the antibacterial rate of 10 % Nisin cement was as high as 85 % after 7 days of aging and almost reached 50 % after 14 days. These positive results sparked our curiosity about whether Nisin cements exhibit antibacterial activity against clinically isolated MRSA. This clinically isolated MRSA strain is extremely drug-resistant, even high-dose gentamicin-loaded cement showed resistance at the 3-day time point (Fig. S9). We found that the antibacterial rate of 10 % Nisin cement reached 66.05 ± 1.15 %, while both 2.5 % and 5 % Nisin cements showed antibacterial rates exceeding 50 % (Fig. S10). AMP can disrupt cell membranes and induce bacterial lysis [[44], [45], [46]]. In addition, nisin exerts its antibacterial effect by interacting with the cell wall precursor lipid II, thereby inhibiting peptidoglycan synthesis while simultaneously forming highly specific pores [47]. SEM was used to observe the morphology of bacteria on different bone cements. As shown in Fig. 3E, *S aureus* maintained normal spherical morphology on PMMA. In contrast, when bacteria were inoculated onto Nisin cements, their status deteriorated, with cell walls shrinking and deforming (see arrows in Fig. 3E). These results indicate that the surface-immobilized Nisin comonomer exerts destructive effects on bacteria.

### 3.3 Cytotoxicity, hemolytic activity, and mineralization *in vitro*

**Fig. 4:**
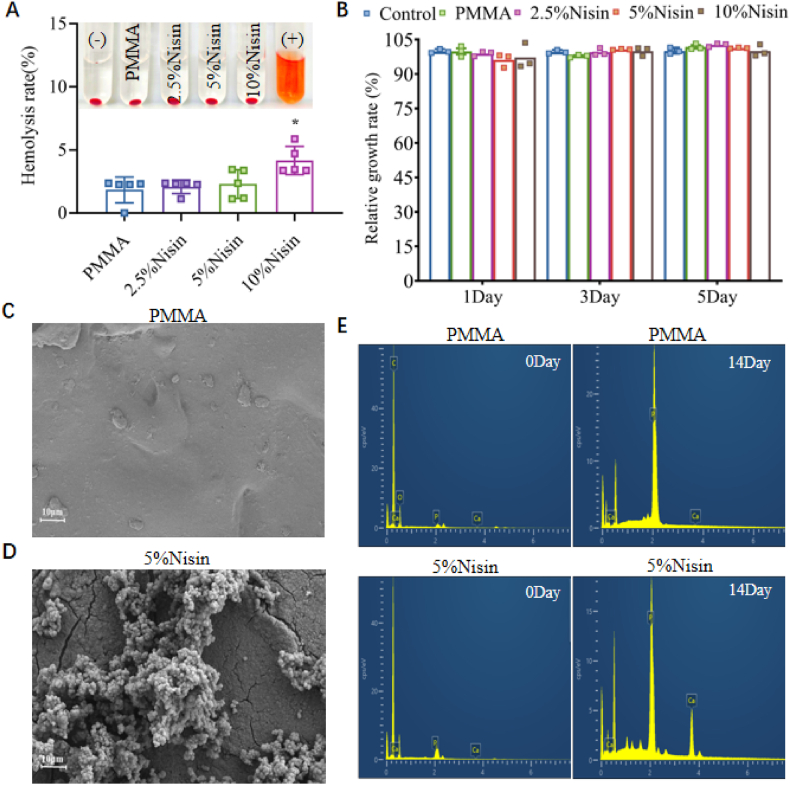
(A) Hemolytic activity of PMMA cement, Nisin cements (∗: p < 0.05). (B) Relative growth rate (RGR) of PMMA cement, Nisin cements. (C) SEM images of PMMA cement after soaking in SBF for 7 days. (D) SEM images of 5 % Nisin cement after soaking in SBF for 7 days. (E) EDS spectrum of PMMA cement and 5 % Nisin cements before and after soaking for 7 days.

Since biocompatibility is a fundamental requirement for biomedical materials, and the introduction of nisin into the cement may potentially increase its toxicity, hemolytic activity and cytotoxicity were selected as evaluation indices for *in vitro* biocompatibility. When comparing the hemolytic activity of PMMA cement and Nisin cements, the hemolytic activity of all cements was found to be lower than 5 %. This indicates that Nisin cements have no hemolytic activity. Then, we evaluate the cytotoxicity of both PMMA cement and Nisin cements in MC3T3-E1 cells by measuring cell viability via CCK-8 assay. There was no significant difference in RGR between the PMMA and Nisin cements (p > 0.05), and both values were greater than 75 %, indicating that the introduction of Nisin did not cause cytotoxicity. During the fixation of joint prostheses or repair of bone defects, the mineralization of bone cement plays a crucial role in the regeneration of new bone tissue and the integration of the cement-bone interface. As shown in Fig. 4C and D, we observed a significant bio-mineralization layer on the surface of 5 % Nisin cement in SEM. As shown in Fig. 4E, the EDS spectrum also showed increased peaks of P and Ca. This may be because Nisin is rich in cationic residues such as lysine and histidine, which can preferentially adsorb phosphate (PO_4_^3−^) from solution and subsequently capture Ca^2+^ to form initial precursor nuclei [48]. In contrast, PMMA cement exhibits trace amounts of Ca and P signals. When combined with the SEM images, we attribute these signals to residual SBF ions on the surface, which do not constitute evidence of a mineralized layer. Figs. S11 and S12 provides a detailed evaluation of the *in vitro* mineralization performance of the PMMA cement and Nisin cements. These results all show that PMMA cement has no *in vitro* mineralization, while the bone cement with Nisin added has obvious *in vitro* mineralization, and suggesting that Nisin cements exhibit good mineralization activity and serve as excellent biomaterials for promoting bone regeneration.

### 3.4 Osteogenic differentiation *in vitro*

**Fig. 5:**
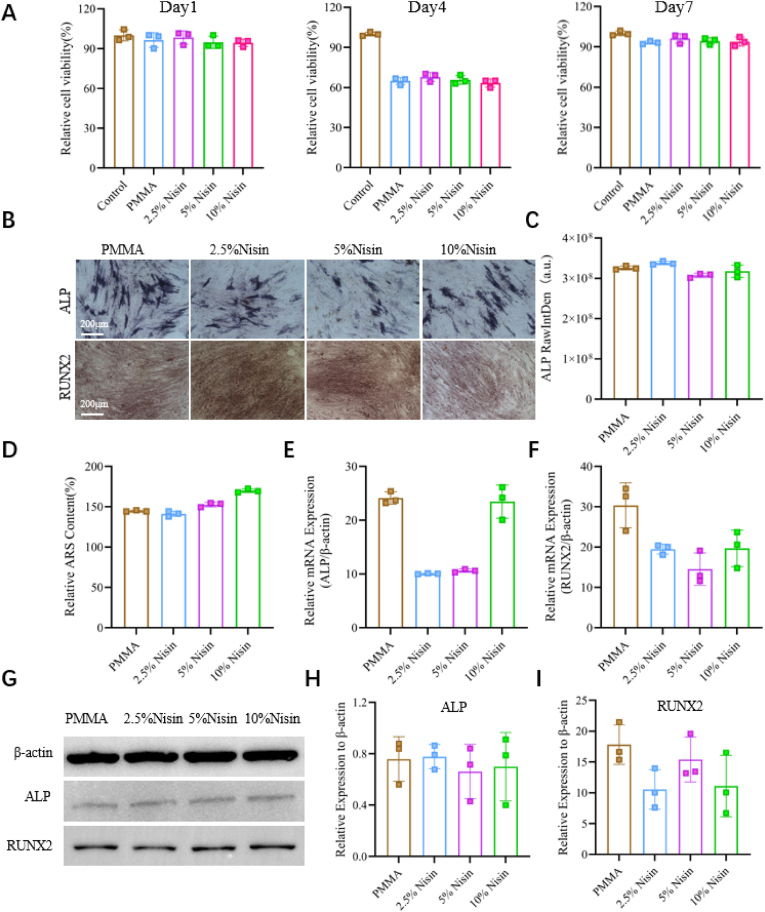
Osteogenic differentiation effect of Nisin cements. (A) RGR of hBMSCs on days 1, 4, and 7 on PMMA and Nisin cements. (B) ALP staining and ARS staining images of hBMSCs on PMMA and Nisin cements. (C) Quantitative analysis of ALP staining. (D) Quantitative analysis of ARS staining. (E, F) Relative gene transcription levels of osteogenic markers (ALP and Runx2) of hBMSCs on day 14. (G–I) Relative protein expression levels of osteogenic markers (ALP and Runx2) detected by Western blotting.

PMMA bone cement is one of the most successful biomaterials in the field of orthopedic fixation [49,50]. It is generally considered that PMMA cement has biological inertness, and it is necessary to study whether the addition of Nisin will cause adverse effects on osteoblasts. In this regard, hBMSCs were used to evaluate the cell adhesion and proliferation on the Nisin cement. We found that on days 1, 3, and 5, all Nisin cements compared with the bare PMMA cement did not cause a decrease in hBMSCs proliferation or cytotoxicity (Fig. 5A, Fig. S13). Subsequently, after 14 days of osteogenic induction, the ALP activity of hBMSCs cultured on PMMA cement and Nisin cements was detected. As shown in Fig. 5B, all cements exhibited ALP activity, and quantitative analysis indicated that the addition of Nisin had no effect on the ALP activity of bare PMMA cement (Fig. 5C). ARS staining was used to evaluate the osteogenic differentiation. The results showed that the bone cement enhanced the mineralization of the cellular matrix after the addition of Nisin, and it was observed that the mineralization was positively correlated with the proportion of Nisin (Fig. 5B and D). qRT-PCR was used to analyze the osteogenic differentiation of hBMSCs.

The gene expression of osteogenesis-related proteins was analyzed, including ALP (an early marker of osteoblast differentiation) and runt-related transcription factor 2 (Runx2, a pivotal transcription factor in regulating osteoblastic function). As shown in Fig. 5E and F, compared with PMMA cement, only the ALP gene expression of 10 % Nisin cement was close to that of PMMA, while the gene expressions of Runx2 and ALP related to osteogenic differentiation all decreased in other groups. This phenomenon may be attributed to low-dose Nisin inhibiting ALP gene transcription. As the Nisin dose increases, this inhibitory effect gradually diminishes, becoming comparable to that of the PMMA group. In contrast, regardless of the Nisin dose, its effect on Runx2 gene transcription remains consistently inhibitory. The protein translation level was inconsistent with the PCR results. The Runx2 expression levels in 2.5 % and 10 % Nisin cements were lower than those in PMMA cement, 5 % Nisin cement showed a Runx2 expression level similar to that of PMMA cement. This may be because Nisin inhibits not only the transcription of the Runx2 gene, but also the translation of the protein. Additionally, we found that the ALP expression in 5 % Nisin cement was slightly reduced, but it can be seen that the ALP expressions of all Nisin cements were similar to that of PMMA cement (Fig. 5G–I). This may be because the timing of gene transcription and protein translation is different. In the late stage of the experiment, the expression of genes decreased and protein translation increased, resulting in that the protein translation level did not change with the change of gene expression, thus leading to the inconsistency between gene transcription results and protein translation results. These results indicate that the addition of Nisin does not promote the osteogenic differentiation of inert PMMA cement. Notably, Nisin does not exert adverse effects on osteogenic differentiation. Nisin does not serve as a universal solution, and this conclusion contributes to a more objective understanding of Nisin's functional scope.

### 3.5 Biocompatibility *in vivo*

**Fig. 6:**
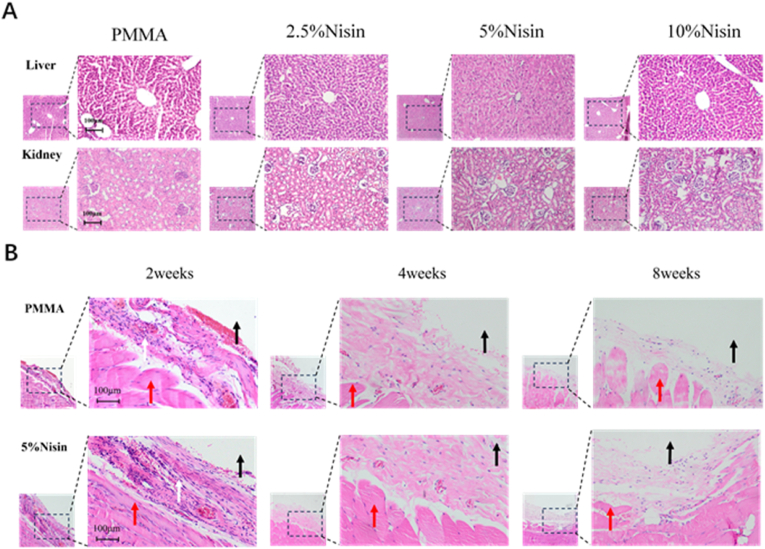
(A) Liver and kidney sections from the short-term acute toxicity test in mice. (B) Tissue sections around the implant in the back muscle implantation test in rats (Black arrows indicate the location of the implant; White arrows indicate inflammatory cells; Red arrows indicate muscle cells).

Bone cement needs to be implanted into the body. Therefore, in addition to *in vitro* cytotoxicity evaluation, we also carried out *in vivo* acute toxicity tests. All mice showed no signs of coma, shock, vomiting, diarrhea, or any other symptoms. All mice showed overall weight gain, and there was no significant difference on the third day (p > 0.05, Table S3). Compared with the saline group, neither PMMA cement nor Nisin cements damaged the normal structure of hepatic lobules and glomeruli, and no inflammatory cells or necrosis of liver nor kidney cells were found (Fig. 6A). These results all indicate that the experimental animals did not exhibit acute toxicity. The *in vivo* biocompatibility of Nisin cements were examined by implanting cements into the backs of rats, and histological analysis demonstrated the tissue response of the muscle surrounding the samples in Fig. 6B. After 2 weeks of implantation, a small number of inflammatory cells were observed in the muscle surrounding area of the bone cement, indicating a mild and early inflammatory response to the muscle tissue after implantation. Compared with that at 2 weeks, the number of inflammatory cells in each group decreased at 4 and 8 weeks, suggesting that the degree of inflammatory response was reduced, which is a positive sign of tissue healing. Nisin cement, like PMMA, has excellent tissue compatibility, and the addition of Nisin does not induce toxicity, which may be attributed to the fact that Nisin cement does not release antibacterial agents, thereby avoiding excessive immune responses, reducing inflammatory reactions, and causing no damage to the liver, kidneys, or muscles in mice.

### 3.6 Antibacterial activity *in vivo*

**Fig. 7:**
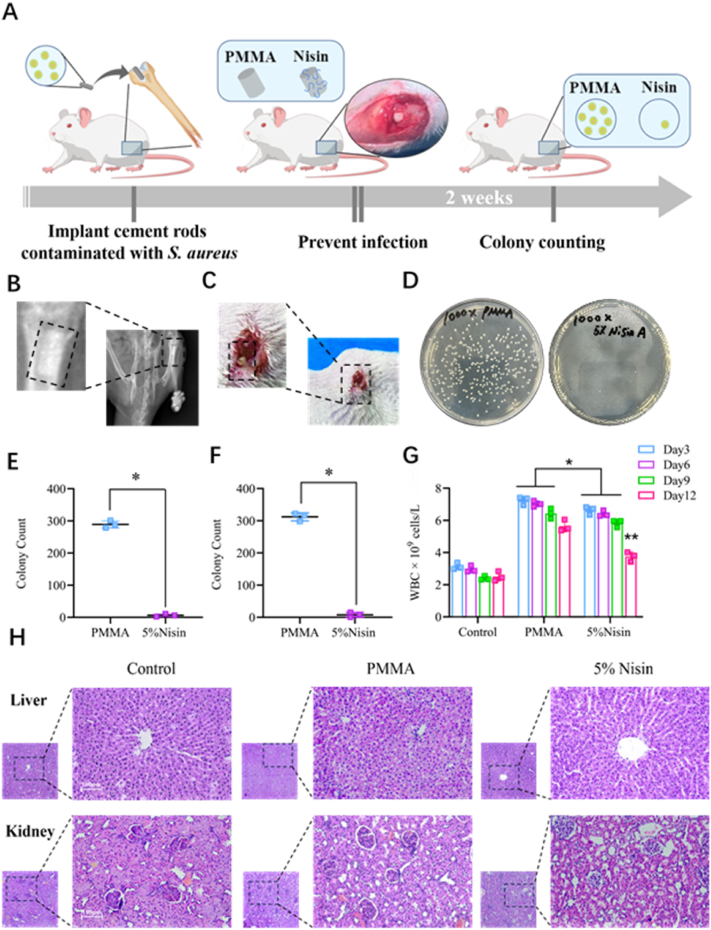
(A) Schematic representation of implant-related infection in rats. (B) X-ray images of the cement implantation site. (C) Manifestations of induced rat infection after 2 weeks. (D) Bacterial counting to evaluate implant-related infection using PMMA cement and 5 % Nisin cement after 2 weeks. (E) Bacterial counting on the surface of the cement. (F) Bacterial counting in the tissues around the cement. (G) WBC counting to evaluate implant-related infection. (H) Pathological examination sections of the liver and kidney after 2 weeks. Generic Diagramming Platform (https://biogdp.com/) was used to draw schematic diagrams.

Since the properties of 5 % Nisin cement are more in line with clinical needs, this formulation was used for subsequent research. Despite the extensive use of antibiotics in the perioperative period, the implant-related infections are serious complications that commonly happen due to local granulocyte defects [51]. Encouraged by the potent antibacterial activity *in vitro* and favorable biosafety of Nisin cement, we continued to evaluate their *in vivo* antibacterial activity in a rat model (Fig. 7A). An implant-related infection model was established utilizing *S. aureus*-contaminated cement rods. Each bone cement was immersed in 1 mL of *S. aureus* bacterial solution at a concentration of 0.5 × 10^8^ CFU/mL for 6 h. Following immersion, the cements were directly implanted into experimental animals (Fig. 7B). After implantation, the rats implanted with PMMA cement exhibited purulent swelling at the implantation site, whereas no such purulent swelling was observed in the implantation site of rats receiving Nisin cement (Fig. 7C). Two weeks later, the rats were sacrificed. To qualitatively evaluate the anti-infective ability of cements, the cement rods were removed from the rat femurs. The rods were subjected to ultrasonic vibration to remove attached bacteria, and then the bacteria were diluted in PBS and cultured on agar plates at 37 °C for 24 h to determine the number of bacterial colonies (Fig. 7D and E). Meanwhile, the tissue around the cement rods was crushed, and the supernatant was collected for culture. The bacteria were diluted in PBS and cultured on agar plates at 37 °C for 24 h to determine the colony count (Fig. 7F). All these results showed that a large number of viable bacteria existed on the surface of PMMA cement and in the surrounding tissues. Conversely, the antibacterial efficiency of both the Nisin cement surface and surrounding tissues is up to 99 %. This result matched the purulent condition observed in Fig. 7C.

White blood cells (WBCs) are inflammatory indicators reflecting bacterial infections. Regular monitoring of WBCs change in rats after implantation of *S. aureus*-contaminated cement rods can reflect the inflammatory status. The normal WBC count in rats typically ranges from 2.3 to 3.4 × 10^9^ cells·L^−1^. Due to implant-related infections, the WBC count in the PMMA group reached 5.4–7.4 × 10^9^ cells·L^−1^. Notably, 12 days after implantation, Nisin cement significantly reduced the rat WBC count to 3.8–4.0 × 10^9^ cells·L^−1^, basically recovering to the normal level (Fig. 7G). Finally, we were curious whether the implantation of Nisin cement for up to 2 weeks would cause toxic effects in major organs (such as the liver and kidneys) of rats. Histological analysis of the anatomical tissues of rat livers and kidneys showed no obvious toxic effects (Fig. 7H). Similar to clinically used PMMA cement without any drug addition, the Nisin cement showed no obvious hepatic and renal toxicity in rats after implantation. Pathological sections showed that the morphological structure of liver lobules was intact, with no obvious inflammatory cell infiltration in the portal area. No obvious structural damage was observed in glomeruli and renal tubules, and there was no inflammatory cell infiltration. These results suggest that Nisin cement is a promising and safe approach for preventing periprosthetic infections.

### 3.7 Demonstration of translational applications

**Fig. 8:**
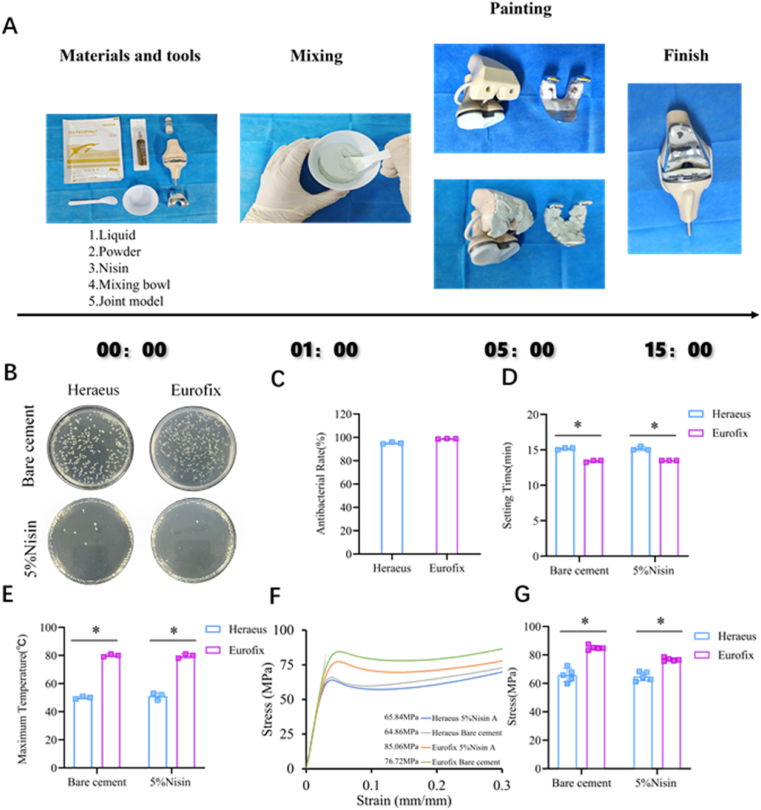
(A) Demonstration of Nisin-based technology in operating room. (B) Bacterial counting was performed to evaluate the antibacterial activity of bare cements and 5 % Nisin-added cements from the two brands. (C) Antibacterial rates of 5 % Nisin-added commercially available cements. (D) Setting time of bare cements and 5 % Nisin-added cements from the two brands (∗: p < 0.05). (E) The maximum temperature of bare cements and 5 % Nisin-added cements from the two brands (∗: p < 0.01). (F) Stress-strain curves of bare cements and 5 % Nisin-added cements from the two brands (the data below the curves is the compressive strength). (G) Compressive strength of bare cements and 5 % Nisin-added cements from the two brands (∗: p < 0.01).

To further demonstrate the translational application of this natural peptide comonomer Nisin-based technology, we used clinically used Heraeus brand bone cement to perform joint prosthesis replacement in the operating room. The process involved the preparation of the natural peptide comonomer Nisin, along with components and tools such as the solid phase, liquid phase, mixing bowl, stirring rod, knee joint model, and joint prosthesis (Fig. 8A). First, the solid phase of the bone cement and Nisin was placed in a bowl; the liquid phase was then added at a solid-to-liquid ratio of 2:1, and the mixture was stirred with a stirring rod for 1 min. The bone cement in the dough stage was transferred to the surface of the joint and joint prosthesis. Subsequently, the prosthesis was implanted and fixed onto the joint surface, and excess cement was removed to complete the prosthesis replacement process. The entire procedure, performed in line with clinical operations and easily completed under aseptic conditions, allows surgeons sufficient time to complete the operation.

This translational demonstration has driven us to eagerly explore the key properties of Nisin-added commercial bone cements, including antibacterial activity, operability, and mechanical strength.

We incorporated 5 % Nisin into clinical bone cements from two brands, and their antibacterial rates were enhanced to over 95 %: specifically, 95.09 ± 0.66 % for Heraeus and 98.92 ± 0.17 % for Eurofix (Fig. 8B and C). The addition of Nisin had almost no impact on the setting time and polymerization temperature (Fig. 8D and E). The mechanical strengths of the two commercial cements with Nisin showed no difference compared to bare cement, indicating that Nisin addition does not affect the mechanical strength of commercial cements (Fig. 8F and G). These results demonstrate that Nisin-based POC technology can be well compatible with commercial bone cements.

Bone cement is not only used for fixing joint prostheses but also widely applied in vertebroplasty. From an application perspective, we aim to expand the horizon of this technology. In vertebroplasty, the injectability, fillability, and anti-leakage properties of bone cement are of great concern. Fig. S15a demonstrates the excellent injectability of Nisin cement. Then, we conducted evaluations using 3D printed model and bovine vertebrae. We injected Nisin cement into a 3D vertebral model filled with foam. After removing the surface foam, the bone cement within the 3D model showed an elliptical shape, indicating good fillability and resistance to leakage (Fig. S15b). In addition, we established a working channel in bovine vertebrae, and through reconstructed computed tomography (CT) images, the good distribution of bone cement in the channel was observed (Fig. S15c). The study on bovine vertebrae further confirmed that Nisin bone cement has excellent injectability and fillability.

### 3.8 Limitations

1. The natural peptide-based POC technology for antibacterial bone cement remains in the developmental stage, with critical clinical factors affecting its performance (e.g., mixing process, surgical field contaminants, deviations in binary component proportions) yet to be evaluated.
2. Although *in vivo* studies have been conducted to address the research needs, long-term investigations into the mechanical and biological stability of the cement should be performed in future work.
3. In principle, this technology holds potential universal applicability (e.g., in dentistry, vertebroplasty), but systematic studies to verify such universality are required in subsequent research.

## 4 Conclusion

In recent years, bone repair materials have advanced rapidly, with examples including bioactive glass (BG), calcium phosphate cement (CPC), PMMA bone cement, magnesium phosphate cement (MPC), etc [[52], [53], [54], [55], [56], [57], [58], [59], [60], [61], [62], [63], [64], [65], [66], [67]]. Among these, PMMA bone cement remains a cornerstone material in orthopedic fixation. A series of exciting advancements have been pursued, which further demonstrates the vitality and promising prospects of PMMA cement—this, in turn, positions it as an ideal candidate for addressing infection management challenges in clinical orthopedics. Notably, antibiotic-loaded PMMA bone cement (ALBC), as a key form of this material, is extensively employed in virtually standard workflows [1,4,[68], [69], [70], [71], [72]] However, issues such as the burst release of antibiotics and biological toxicity undermine the efficacy of ALBC in preventing infections [11,13,20,72]. Promising investigations have validated that covalently integrating therapeutic agents within the cement matrix offers a highly efficient antibacterial strategy-without compromising its mechanical integrity. Yet, the translation of these breakthrough approaches into clinical practice is hindered by their reliance on sophisticated fabrication techniques.

In this study, we report the synergistic engineering of a natural peptide comonomer via POC technology to develop Nisin cement. This cement demonstrates mechanical properties compliant with ASTM standards, potent anti-*S. aureus* activity, and significantly enhanced *in vitro* biomineralization capacity. Notably, while *in vitro* and *in vivo* analyses confirmed its excellent biocompatibility, the unexpected boost in biomineralization prompted us to explore potential osteogenic effects-despite osteogenesis not being the primary design objective. Although Nisin did not elicit dramatic osteogenic enhancement, our findings unequivocally show no adverse impact on PMMA's osteogenic activity. *In vivo* infection models further validated its efficacy in preventing implant-associated infections, while compatibility studies demonstrated seamless integration with commercial bone cements-a particularly promising attribute from a translational perspective. Overall, this POC technology empowers clinicians to fabricate bone cement with optimized antibacterial potency, mechanical strength, and biocompatibility-*without specialized training*-thereby holding transformative potential for standard surgical workflows. Concurrently, the data presented in this study have the potential to substantially reduce unnecessary antibiotic administration and advance global antibiotic stewardship efforts.

## CRediT authorship contribution statement

**Yang Xu:** Writing – review & editing, Writing – original draft, Visualization, Supervision, Project administration, Methodology, Funding acquisition, Data curation, Conceptualization. **Hao Lin:** Visualization, Methodology, Data curation. **Yin-Yu Qi:** Writing – original draft, Visualization, Methodology, Formal analysis, Data curation. **Chen Wang:** Visualization, Data curation. **Long-Xu Han:** Visualization, Data curation. **Fang He:** Writing – original draft, Methodology, Funding acquisition. **Hong-Xun Sang:** Writing – original draft, Funding acquisition, Data curation. **Jian-Jun Chu:** Writing – review & editing, Writing – original draft, Supervision, Methodology, Funding acquisition.

## Ethics statement

The animal study was approved by this study was conducted with approval from the Biomedical Ethics Committee of Hefei University of Technology (No. HFUT20241025-002).

## Declaration of competing interest

The authors declare that they have no known competing financial interests or personal relationships that could have appeared to influence the work reported in this paper.

## Data Availability

Data will be made available on request.
