# Supplementary material for: Point-of-care bone cement based on natural peptide comonomer protects orthopaedic implants from bacterial challenge
Source: Mater Today Bio. 2025 Oct 7;35:102388. doi: 10.1016/j.mtbio.2025.102388 (PMC12538693; doi:10.1016/j.mtbio.2025.102388)
Supplement: Multimedia component 1 [file mmc1.docx]

**Supplementary information**

**Point-of-care bone cement based on natural peptide comonomer protects orthopaedic implants from bacterial challenge**

Yang Xu ^1,2^, Hao Lin ^3,4^, Yin-Yu Qi ^2^, Chen Wang ^1,2^, Long-Xu Han ^2^, Fang He ^1,3^, Hong-Xun Sang ^1,4^ and Jian-Jun Chu ^1,2^

^1.^ School of Food and Biological Engineering, Hefei University of Technology, Hefei, China.
^2.^ The Second People's Hospital of Hefei, Anhui Medical University, Hefei, China.
^3.^ Hefei BOE Hospital, Shanghai University Medical College, Hefei, China.

^4.^ Shenzhen Hospital, Southern Medical University, Shenzhen, China.

These authors contributed equally: Yang Xu, Hao Lin, Yin-Yu Qi.

Correspondence authors: chujianj@mail.ustc.edu.cn (Jian-Jun Chu), [hefang8@126.com](mailto:hefang8@126.com) (Fang He), [xuyang@hfut.edu.cn](mailto:xuyang@hfut.edu.cn) (Yang Xu).

**Supplementary Information contains:**

Supplementary Tables S1−S3

Supplementary Figures S1−S15

**Table S1.** Resistance spectrum of clinically isolated MRSA. The strain is MRSA and is resistant to a variety of antibiotics, including Clindamycin and Gentamicin, which are antibiotics approved by the FDA for use in bone cement.

| Bacteria | Methicillin-resistant *Staphylococcus aureus* (MRSA) | | |
| --- | --- | --- | --- |
| Drug | Measured value | Methodology | Result |
| Penicillin | ≥0.5 | MIC | **+** |
| Oxazole penicillin | ≥4 | MIC | **+** |
| Erythromycin | ≥8 | MIC | **+** |
| **Clindamycin** | ≥8 | MIC | **+** |
| **Gentamicin** | ≥16 | MIC | **+** |
| Levofloxacin | 1 | MIC | **-** |
| Moxifloxacin | ≤0.25 | MIC | **-** |
| Tetracycline | ≤1 | MIC | **-** |
| Tigecycline | ≤0.12 | MIC | **-** |
| Linezolid | 2 | MIC | **-** |
| Vancomycin | 1 | MIC | **-** |
| Cotrimoxazole | ≥320 | MIC | **+** |
| Rifampicin | ≤0.5 | MIC | **-** |
| Nitrofurantoin | 32 | MIC | **-** |

**Table S2.** The primer sequences of genes in qRT-PCR

| Gene | 5′-3′ | Primers |
| --- | --- | --- |
| *ALP* | forward | GGGGTCAAAGCCAACTACAA |
|  | reverse | CTTCCCTGCTTTCTTTGCAC |
| *RUNX2* | forward | GCCGGGAATGATGAGAACTA |
|  | reverse | GCCGGGAATGATGAGAACTA |
| *β-actin* | forward | TGGCACCCAGCACAATGAA |
|  | reverse | CTAAGTCATAGTCCGCCTAGAAGCA |

**Table S3.** Change in body weight of mice within 3 days after intraperitoneal injection (n = 3) (x ± s, g) (*. p < 0.05, compared to after 24 hours of 10%Nsin treatment; **. p < 0.01, compared between 24 and 48 hours after 2.5% and 5%Nisin treatment; ^. p < 0.05, compared between control and 5%Nisin after 48 hours of treatment.).

|  | 24h | 48h | 72h |
| --- | --- | --- | --- |
| control  PMMA  2.5%Nisin A  5%Nisin A  10%Nsin A | 0.387±0.034  0.367±0.080  0.557±0.205  0.360±0.262  0.403±0.225 | 0.277±0.059**^^^**  0.323±0.118  0.023±0.267^**^  -0.110±0.065^**^  -0.023±0.078^*^ | 0.393±0.087  0.410±0.118  0.197±0.094  0.177±0.127  0.260±0.091 |

**Figure S1.** (A) The chemical structure of Nisin, with Dha residues marked in red. (B) HPLC of Nisin monomer before and after heat treatment.


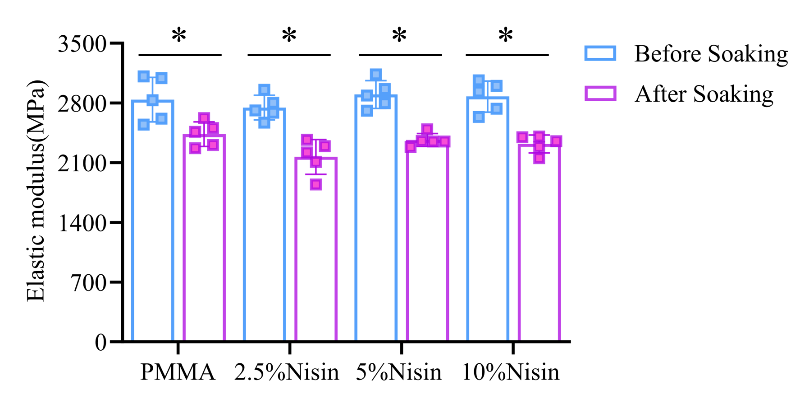


**Figure S2.** Elastic modulus of PMMA cement, 2.5%, 5% and 10% Nisin cement (*. p < 0.01).


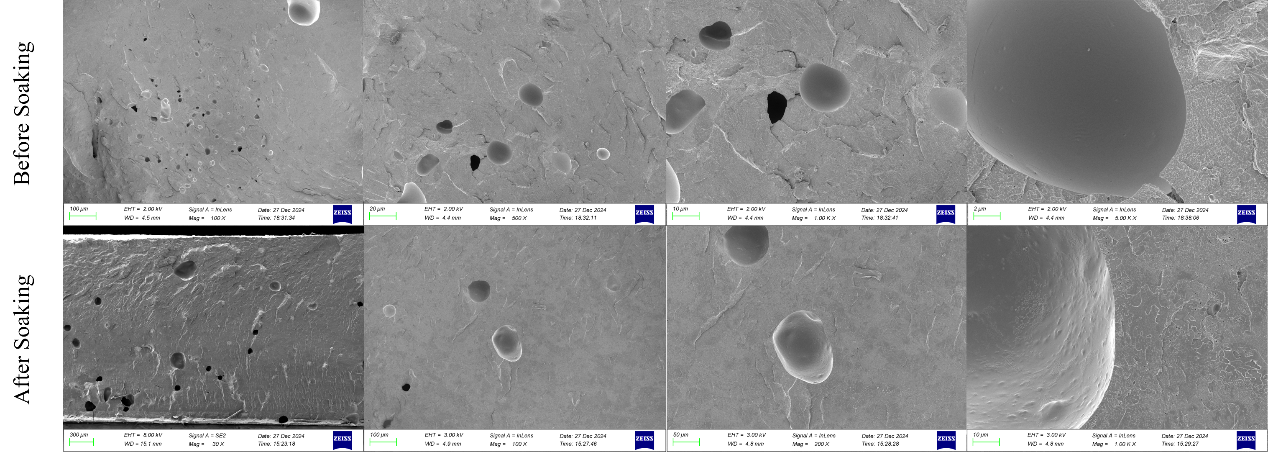


**Figure S3.** SEM image of the fracture section of PMMA bone cement before and after soaking at different magnification fields.


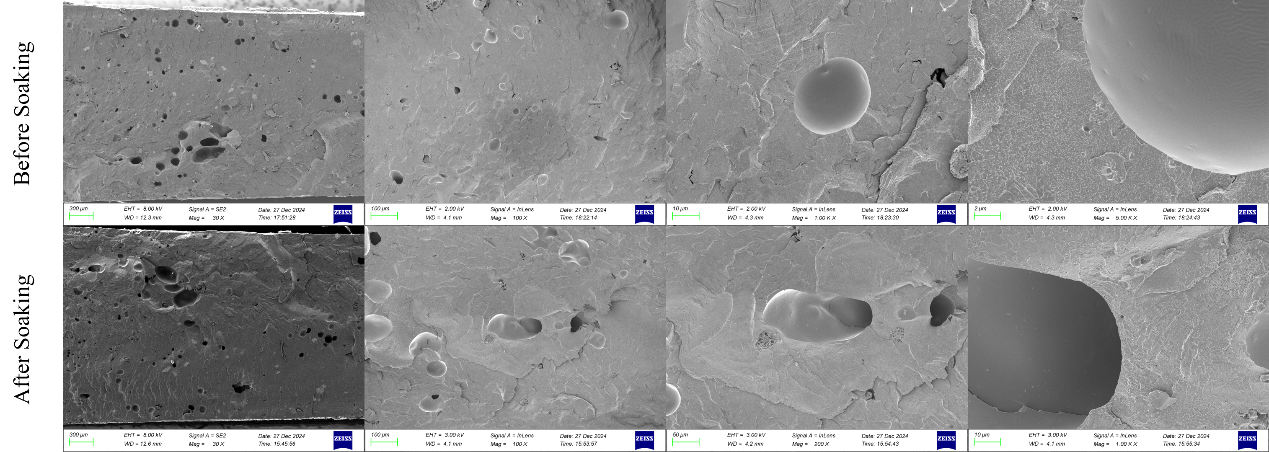


**Figure S4.** SEM image of the fracture section of 2.5% Nisin bone cement before and after soaking at different magnification fields.


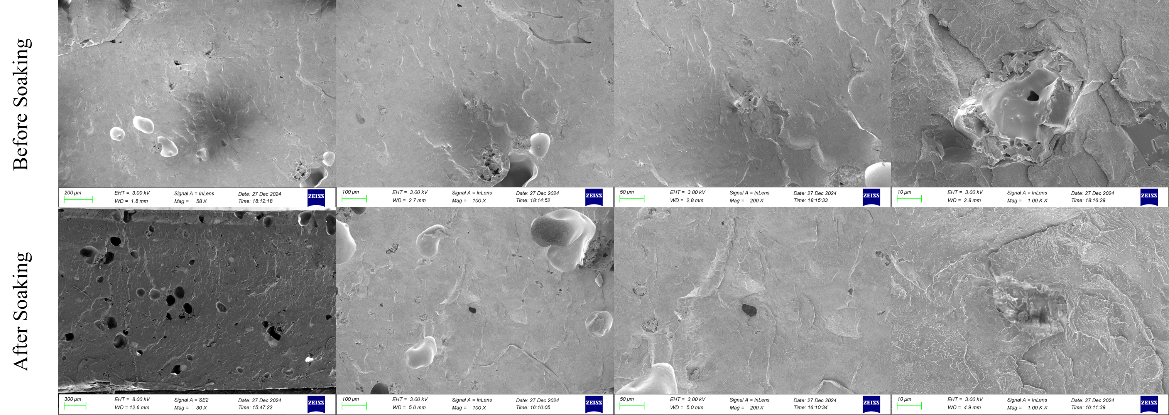


**Figure S5.** SEM image of the fracture section of 5% Nisin bone cement before and after soaking at different magnification fields.


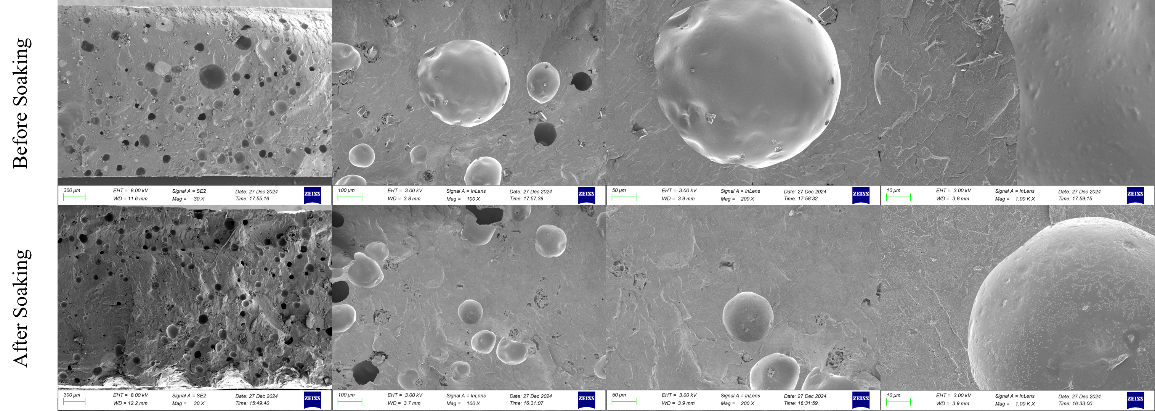


**Figure S6.** SEM image of the fracture section of 10% Nisin bone cement before and after soaking at different magnification fields.


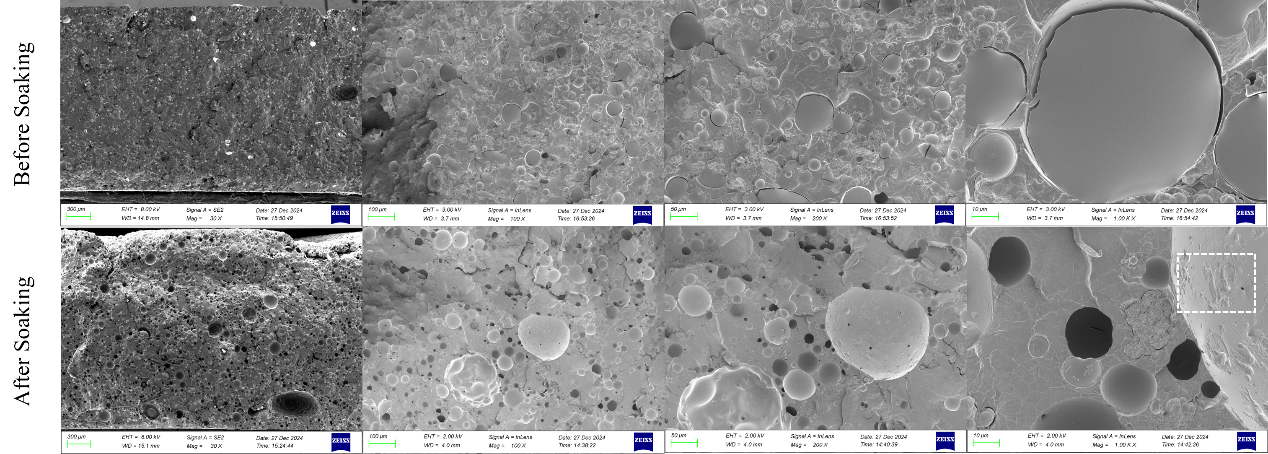


**Figure S7.** SEM image of the fracture section of 5% GS bone cement before and after soaking at different magnification fields. Among them, the white circled area is the residual trace of gentamicin after elution.


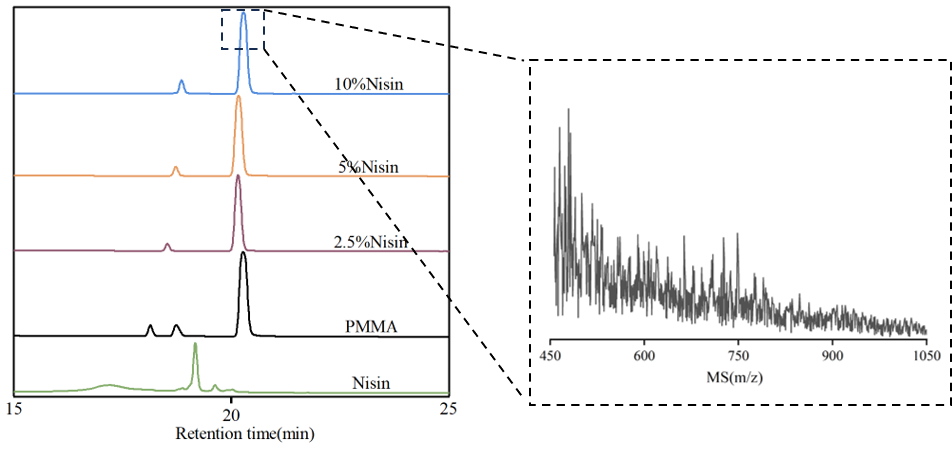


**Figure S8.** HPLC and MS of Nisin, PMMA cement extract, 2.5%, 5% and 10% Nisin cements extract. The retention time of the peak in the dashed box is 20.27 min.


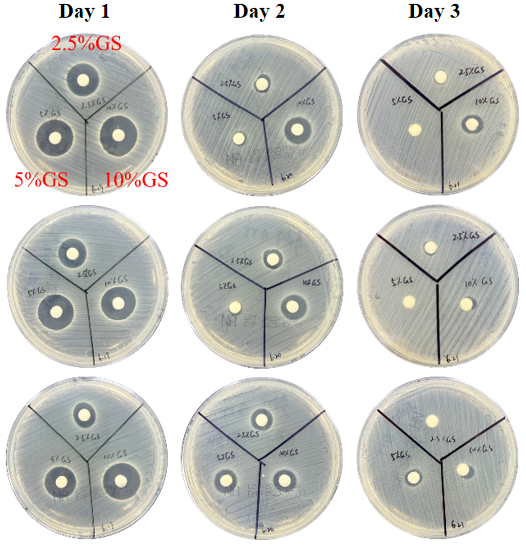


**Figure S9.** Antibacterial activity of 2.5%, 5%, and 10% GS bone cements against clinical MRSA.


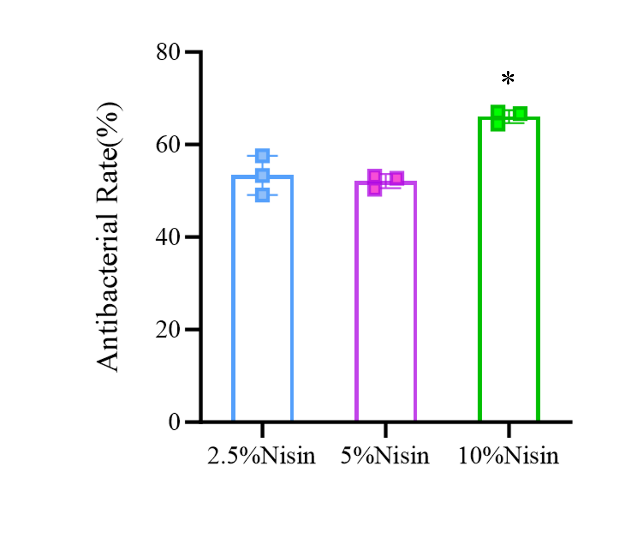


**Figure S10.** Antibacterial rate of 2.5%, 5% and 10% Nisin cements against clinical MRSA (*. p < 0.01).


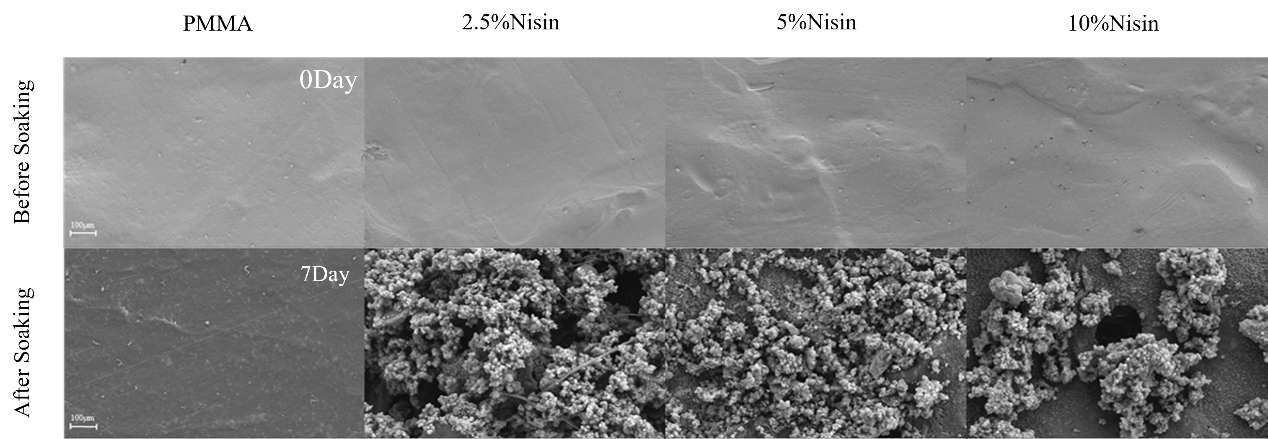


**Figure S11.** SEM images of PMMA cement, 2.5%, 5% and 10% Nisin cement before and after soaking in SBF for 7 days.


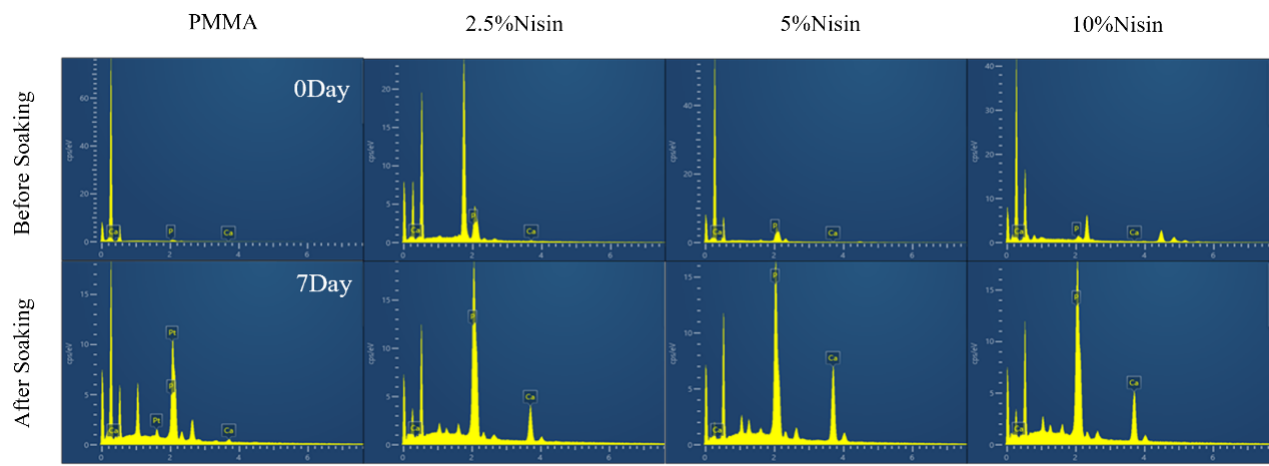


**Figure S12.** EDS spectrum of PMMA cement, 2.5%, 5% and 10% Nisin cement before and after soaking in SBF for 7 days.


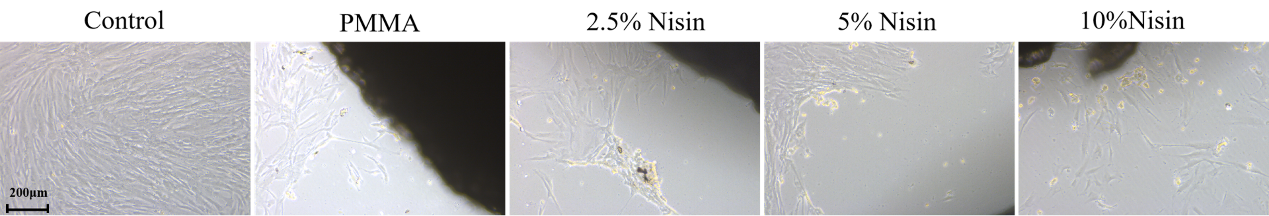


**Figure S13.** hBMSCs were cultured in a specialized medium for 4 days in MTT experiment.


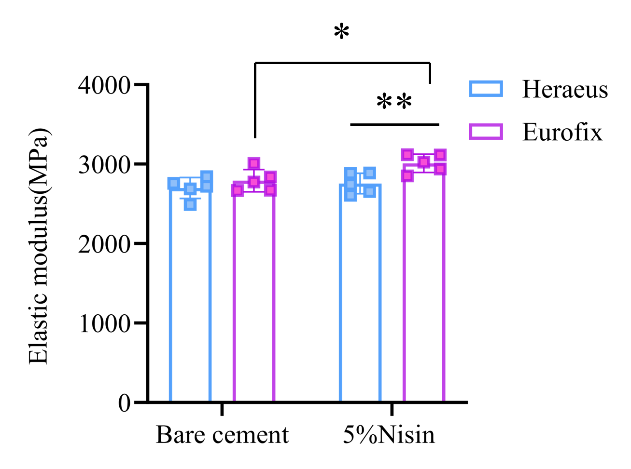


**Figure S14.** Elastic modulus of two kinds of bare commercially available bone cements and those with 5% Nisin added. (*. p < 0.05; **. p < 0.01).


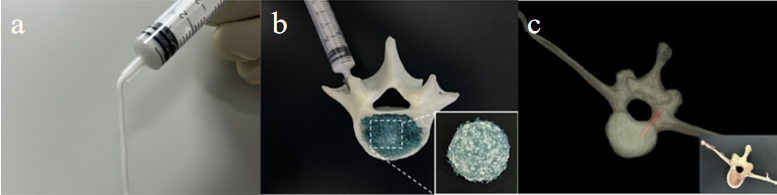


**Figure S15.** Application study of Nisin cement. (a) Injectability of Nisin cement; (b) the filling and leakage performance of Nisin cement was evaluated using a 3D-printed human vertebral body model; (c) the bovine vertebrae were reconstructed by CT after injection of Nisin cement.
